# Supplementary material for: Trends and determinants of acute respiratory infection symptoms among under-five children in Cambodia: Analysis of 2000 to 2014 Cambodia demographic and health surveys
Source: PLOS Glob Public Health. 2023 May 3;3(5):e0001440. doi: 10.1371/journal.pgph.0001440 (PMC10155954; doi:10.1371/journal.pgph.0001440)
Supplement: Attachment — Submitted filename: Response to Reviewers.docx [file pgph.0001440.s001.docx]

**Authors’ point-by-point responses to the editor and reviewers**

CC: atulvashist@thsti.res.in, atulvashist1980@gmail.com

**PGPH-D-22-01879**

Trends and determinants of Acute Respiratory Infection symptoms among Under-five children in Cambodia: Analysis of 2000 to 2014 Cambodia Demographic and Health Surveys

**PLOS Global Public Health**

**Journal Requirements:**

1. PLOS Global Public Health does not copy edit accepted manuscripts (https://journals.plos.org/globalpublichealth/s/criteria-for-publication#loc-5). To that effect, please ensure that your submission is free of typos and grammatical errors.

Dr. Erik Karlsson has revised and made corrections grammatically and accordingly as suggested.

2. We have noticed that you have uploaded Supporting Information files, but you have not included a list of legends. Please add a full list of legends for your Supporting Information files after the references list.

Thank you. We did correct a mistake.

3. In the online submission form, you indicated that your data will be submitted to a repository upon acceptance. We strongly recommend all authors deposit their data before acceptance, as the process can be lengthy and hold up publication timelines. Please note that, though access restrictions are acceptable now, your entire data will need to be made freely accessible if your manuscript is accepted for publication. This policy applies to all data except where public deposition would breach compliance with the protocol approved by your research ethics board. If you are unable to adhere to our open data policy, please kindly revise your statement to explain your reasoning and we will seek the editor's input on an exemption. Please be assured that, once you have provided your new statement, the assessment of your exemption will not hold up the peer review process.

Thank you! We have provided a new statement as an online submission form and highlighted it in the revised manuscript under the Subheading **‘Availability of data’**

“Our study used 2000, 2005, 2010, and 2014 Cambodia Demographic and Health Survey datasets. The CDHS data used are publicly available to the DHS Program website at https://www.dhsprogram.com/data/dataset_admin”.

Additional Editor Comments (if provided):

[Note: HTML markup is below. Please do not edit.]

Reviewers' comments:

Reviewer's Responses to Questions

Comments to the Author

1. Does this manuscript meet PLOS Global Public Health’s publication criteria? Is the manuscript technically sound, and do the data support the conclusions? The manuscript must describe methodologically and ethically rigorous research with conclusions that are appropriately drawn based on the data presented.

Reviewer #1: Partly

Reviewer #2: Yes

Thank you!

2. Has the statistical analysis been performed appropriately and rigorously?

Reviewer #1: Yes

Reviewer #2: No

We agree with the suggestion and it has been revised accordingly.

3. Have the authors made all data underlying the findings in their manuscript fully available (please refer to the Data Availability Statement at the start of the manuscript PDF file)?

The PLOS Data policy requires authors to make all data underlying the findings described in their manuscript fully available without restriction, with rare exception. The data should be provided as part of the manuscript or its supporting information, or deposited to a public repository. For example, in addition to summary statistics, the data points behind means, medians and variance measures should be available. If there are restrictions on publicly sharing data—e.g. participant privacy or use of data from a third party—those must be specified.

Reviewer #1: Yes

Reviewer #2: Yes

Thank you!

4. Is the manuscript presented in an intelligible fashion and written in standard English?

PLOS Global Public Health does not copyedit accepted manuscripts, so the language in submitted articles must be clear, correct, and unambiguous. Any typographical or grammatical errors should be corrected at revision, so please note any specific errors here.

Reviewer #1: Yes

Reviewer #2: Yes

Thank you!

5. Review Comments to the Author

Please use the space provided to explain your answers to the questions above. You may also include additional comments for the author, including concerns about dual publication, research ethics, or publication ethics. (Please upload your review as an attachment if it exceeds 20,000 characters)

Reviewer #1: Manuscript is well written and respects scientific norms. It is also of public health health importance but as mentioned by the authors under limitations, there are worries about the relevance of how the conclusions of the research could be exploited because probably they might not reflect what current epidemiological data would reveal.

Thank you!

We agreed and put as limitations of the study. However, the greatest strength of the study is that assessed the trends of ARIs symptoms among children under 5 years over 15 years. And it used a nationally representative sample that made the findings of the study valid for Cambodian children under 5 years population. Although the findings of the study are similar to the findings of other international studies of the same population, the result can be used as a **base-line value** for comparison in the future.

Furthermore, authors need to check the interpretation of AOR"s and the confidence intervals so that the comments tally with the variables analyzed.

Thank you! We agree with the suggestion and it has been revised accordingly.

The grammar also needs to checked. Please, see the attached tracked copy for comments and edits.

Dr. Erik Karlsson has revised and made corrections grammatically and accordingly as suggested.

Reviewer #2: In this manuscript titled “Trends and determinants of Acute Respiratory Infection symptoms among Under-five children in Cambodia: Analysis of 2000 to 2014 Cambodia Demographic and Health Surveys” the authors have comprehensively discussed about the scenario and prevalence of acute respiratory infection symptoms among the children of age below 5 years. Though the authors have designed the study in an organised way and the sample number of the study is also good, but I have some major concerns listed below.

1. The data presented in the study are very old from 2000-2014. There is no much information about the recent data of the acute respiratory infections in Cambodia. So, I suggest to include the updated information.

Thank you! We revised accordingly. However, recent data on ARIs in Cambodia limited study.

2. Also, the authors should mention the previous studies which linked the prevalence of acute respiratory infections with unhygienic toilet in the discussion section

Thank you! We revised accordingly. Both the literature and discussion sections have been mentioned.

3. It would also be of importance if the authors can include the data of intake of any antibiotics in the last 6 months, which will improve the significance of the study.

Thank you! CDHS did not collect data on the intake of any of the antibiotics in the last 6 months.

It fails into our limitation of the study.

4. Kindly recheck the reference part of the manuscript, it is not in the systematic order.

Revised as suggested.

5. Also, the reduction in the children death may be due to the robust vaccination program, which could be highlighted in the manuscript.

Thanks. We have provided it in the revised manuscript.

6. PLOS authors have the option to publish the peer review history of their article (what does this mean?). If published, this will include your full peer review and any attached files.

Thank you!

Do you want your identity to be public for this peer review? If you choose “no”, your identity will remain anonymous but your review may still be made public.

“Yes”

For information about this choice, including consent withdrawal, please see our Privacy Policy.

Reviewer #1: Yes: Professor Gregory Edie Halle-Ekane (MD, FWACS)

Reviewer #2: Yes: Dr. Deepjyoti Paul

[NOTE: If reviewer comments were submitted as an attachment file, they will be attached to this email and accessible via the submission site. Please log into your account, locate the manuscript record, and check for the action link "View Attachments". If this link does not appear, there are no attachment files.]

While revising your submission, please upload your figure files to the Preflight Analysis and Conversion Engine (PACE) digital diagnostic tool, https://pacev2.apexcovantage.com/. PACE helps ensure that figures meet PLOS requirements. To use PACE, you must first register as a user. Registration is free. Then, login and navigate to the UPLOAD tab, where you will find detailed instructions on how to use the tool. If you encounter any issues or have any questions when using PACE, please email PLOS at figures@plos.org. Please note that Supporting Information files do not need this step.
